# Outcomes after flow diversion for ruptured blood blister-like and dissecting aneurysms: A single-center series

**DOI:** 10.1177/15910199251398360

**Published:** 2025-11-28

**Authors:** William W Wroe, Hussein A Zeineddine, Si Y Yu, Bridger H Freeman, Richard S Cook, Charles M Christensen, Peng Roc Chen, Sunil A Sheth, Spiros L Blackburn

**Affiliations:** 1 Vivian L. Smith Department of Neurosurgery, 12339 McGovern Medical School, The University of Texas Health Science Center at Houston, TX, USA; 2 Department of Neurology, 12340 McGovern Medical School, The University of Texas Health Science Center at Houston, TX, USA

**Keywords:** Flow diversion, pipeline, aneurysm, digital subtraction angiography, short-term outcomes

## Abstract

**Background:**

Flow diverter (FD) use has become a popular method for treating intracranial aneurysms, but evidence in acutely ruptured blood blister-like and dissecting morphologies is limited. Furthermore, there is little evidence regarding the role of short-term follow-up imaging to assess aneurysm stability. This study is a single-center retrospective review of dissecting aneurysms treated by FD, with an analysis of short-term follow-up angiograms to guide post-treatment management.

**Methods:**

Our single-center retrospective review included all patients from 2016 to 2024 with spontaneous subarachnoid hemorrhage from blood blister-like and dissecting aneurysms who underwent treatment with an FD. Outcomes included re-rupture, treatment failure, re-treatment, aneurysm morphological changes, and ischemic/hemorrhagic events. Short-term, inpatient angiograms were used to assess early response to treatment, and long-term follow-up angiogram, >4 months, was used to assess long-term aneurysm obliteration.

**Results:**

Twenty-eight patients underwent FD for treatment of their ruptured dissecting aneurysm. Twelve patients (43%) were treated with coiling plus FD. Treatment failure occurred in four patients (14%). Placement of additional FDs in a second procedure occurred in three patients (11%). Thromboembolic events occurred in five patients (18%). Hemorrhagic complications, not including aneurysm re-rupture, occurred in six patients (21%). Short-term aneurysm improvement occurred in 17 patients (63%). Thirteen patients had long-term follow-up, with 11 (85%) demonstrating complete occlusion.

**Conclusions:**

Flow diversion is a reasonable technique for treating ruptured dissecting aneurysms. Risks are moderate and include short-term aneurysm growth and re-rupture. Early post-treatment angiography is suggested to help identify aneurysm growth and the need for re-treatment.

## Introduction

Following an aneurysmal subarachnoid hemorrhage (SAH), aneurysm re-rupture is the leading cause of death if left untreated. The rate of aneurysm re-rupture was found in earlier studies to be between 4% and 13.6% the first day, with a cumulative risk of 15% to 20% within the first two weeks,^1,2^ and an associated mortality rate between 20% and 60%.^3^ However, it is known that the re-rupture rate is much higher for dissecting and blood blister-like aneurysms, with rates estimated to be as high as 40% to 71.4% in the first day.^4,5^

Open surgical clipping and endovascular coiling are well-established methods of occluding aneurysms, thereby “securing” them from re-rupture. However, not all aneurysms are amenable to occlusion by these traditional techniques. Dissecting and blood blister-like aneurysms are characterized by a fragile wall, an indistinct neck, and a longitudinal shape, making clipping or coiling techniques challenging.^6,7^

Following the pipeline for uncoilable or failed aneurysms trial,^8,9^ the role of flow diverters has expanded significantly, with many centers experiencing success with use across a variety of locations and morphologies, reporting occlusion rates generally >70% at 6-month follow-up and progressively higher thereafter.^10,11^

Because previous large studies primarily contain only non-ruptured aneurysms, the role of flow diverter (FD) in acutely ruptured aneurysms is less established. Additionally, only a few studies have independently considered ruptured blood blister-like and dissecting morphologies,^12,13^ and it is unclear when these aneurysms can be declared “secured” after FD placement. Published studies have shown that re-rupture after treatment is possible, but there is limited data regarding short-term, inpatient imaging, which can be critical for assessing the degree of aneurysm occlusion in this dynamic pathology. This ambiguity has important consequences on SAH management, such as blood pressure control, cerebrospinal fluid diversion, and intrathecal or intra-arterial antispasmodics.

At our institution, an early post-treatment repeat diagnostic subtraction angiography (DSA) is routinely obtained on all SAH patients, typically on post-bleed day (PBD) 7, to evaluate for aneurysm occlusion, identify radiographic vasospasm, and guide management of the previously mentioned parameters. For aneurysms treated with FDs, we often perform this follow-up DSA earlier. Here, we evaluate our institution's outcomes after FD for ruptured aneurysms and whether the use of early post-treatment DSA is helpful in determining the need for further treatment.

## Methods

### Study population

**Figure 1.**
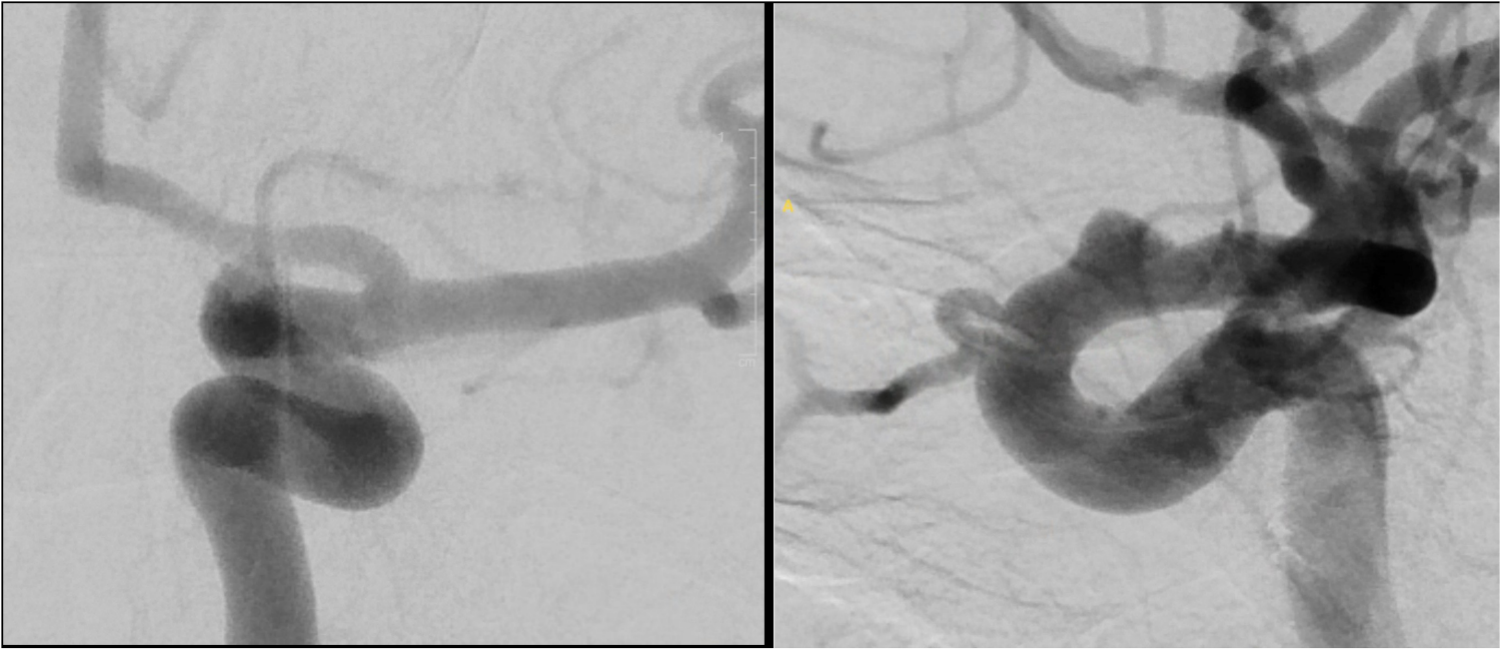
DSA of the left internal carotid artery showing a classical dorsal wall, ophthalmic segment, blood blister-like aneurysm. (Right) AP view; (Left) lateral view.

DSA: diagnostic subtraction angiography; AP: anterior posterior.

This is a single-center retrospective review of all patients with SAH due to a ruptured intracranial dissecting or blood blister-like aneurysm who were treated with FD between the dates of February 2016 and December 2024. Four aneurysms in our cohort met the strict description of blood blister-like aneurysms. That is, those aneurysms located on the dorsal aspect of the ophthalmic segment of the internal carotid artery (ICA) (Figure 1), also called dorsal variant ophthalmic aneurysms. However, as the terms blister and blood blister-like aneurysm are often used in the literature interchangeably with dissecting aneurysm, and no well-defined distinction exists, all these aneurysms were grouped and labeled as dissecting, and no further distinction is made in this paper except for a brief summary in the ‘Results: Blood blister-like aneurysms’ section.

A prospectively maintained database of SAH patients was searched to identify ruptured aneurysms treated with FD. Patients with both anterior and posterior circulation aneurysms were included, as were patients treated with FD alone and FD + coiling.

This study was approved by the institutional review board (IRB) (HSCMS220870), and informed consent of study patients was waived due to the retrospective nature of the data. The study was conducted in accordance with the Declaration of Helsinki and was reported following the Strengthening the Reporting of Observational Studies in Epidemiology guidelines.

### Data collection

On subsequent follow-up DSA, the degree of occlusion was measured using the O'Kelly Marotta score.^14^ This grading score was specifically designed to evaluate outcomes after FD and is based on the degree of angiographic filling and contrast stasis in the aneurysm. Aneurysms were considered improved if they had either decreased visible filling or if they remained stable in size but had a significant increase in stasis. Aneurysms were considered stable if the degree of filling and stasis was the same as before FD placement, and worse if there was an increased aneurysm volume. Images were retrospectively reviewed and graded for aneurysm outcome by two fellowship-trained endovascular neurosurgeons. Blinding was not feasible given the clear evidence of treatment type seen on the DSA.

Radiographic outcomes were recorded for the treatment DSA and all subsequent DSAs, including those done at outpatient follow-up. Short-term follow-up DSA was defined as those done during initial admission, maximum length of stay was 40 days. Our standard inpatient imaging strategy has evolved as our experience with FD for ruptured aneurysms has grown, and it is now the standard to do a repeat DSA 3 to 4 days after initial FD placement. Additional inpatient imaging is then decided upon based on this result. Our standard follow-up imaging regimen for ruptured aneurysms treated with FD is to repeat a DSA 3 to 6 months after discharge, with further outpatient imaging based on the result. For this study, long-term follow-up outcome was defined as the results of the last DSA done that was completed at least 4 months after initial treatment.

Clinical outcomes recorded included aneurysm re-rupture, thrombosis/thromboembolic events, hemorrhagic complications, and patient status at discharge. FD treatment failure without re-rupture was defined as either enlargement in size or length without re-rupture. Data on antiplatelet use were also collected.

## Results

A total of 28 patients underwent flow diversion within the specified period for treatment of their ruptured dissecting aneurysm. The mean age was 54.8, with 22 (79%) female patients. Of the ruptured aneurysms targeted, 13 were initially treated with a single FD only, three with two FDs, and 12 with a combination of coiling and a single FD placement. For three patients who received two FDs at the time of initial treatment, one was due to the imperfect placement of the initial FD, which did not cover the entire length of the diseased ophthalmic segment. For the additional two cases, the aneurysm did not show evidence of stasis after placement of the first FD, and the treating neurointerventionalist decided that the benefits of increased coverage of the aneurysm outweighed the thromboembolic risks of two FDs. The average time from aneurysm rupture to initial FD placement was 3.3 days. Additional baseline clinical and radiographic data are given in Table 1.

**Table 1.** Patient characteristics.

Age, average (range)	54.8 years (33–75)
Sex, no. (%)	
Female	22 (78.6)
Male	6 (21.4)
Modified Fisher Grade, no. (%)	
0	1 (7.1)
I	1 (7.1)
II	1 (3.6)
III	10 (35.7)
IV	15 (53.6)
Hunt and Hess Grade, no. (%)	
I	1 (3.6)
II	8 (28.6)
III	8 (28.6)
IV	10 (35.7)
V	1 (3.6)
Aneurysm size, average (mm)	5.8
Aneurysm location, no. (%)	
ICA	6 (21.4)
OA	8 (28.6)
M1	1 (3.6)
ACA	1 (3.6)
PCA	2 (7.1)
BA	3 (10.7)
VA	7 (25.0)
Days from rupture to FD treatment, average (range)	3.29 (0–13)
Initial treatment type, no. (%)	
Single flow diverter	13 (46.4)
Two flow diverters	3 (10.7)
Flow diversion and coiling	12 (42.9)

ICA: internal carotid artery; OA: ophthalmic artery; MI: M1 segment of the middle cerebral artery; ACA: anterior cerebral artery; PCA: posterior cerebral artery; BA: basilar artery; VA: vertebral artery; FD: flow diverter.

### Short-term angiographic changes after FD placement

Twenty-seven (96%) of the patients underwent repeat DSA after FD placement during the same hospitalization, with a median time of 4 days from initial FD placement. As discussed further, one patient re-ruptured 9 h after FD placement and is not included in this group.

The first repeat DSA after FD showed aneurysm improvement in 17 (63%). Aneurysm size and morphology did not change in seven (26%) of the patients on repeat DSA, and two of these patients had an additional FD placed to further reduce aneurysm flow. Three (11%) patients had aneurysm growth on subsequent DSA, with one re-rupturing and two requiring a second FD. A summary of angiographic results is shown in Table 2.

**Table 2.** Angiographic results after treatment with flow diverter (FD).

Short-term angiographic results, no. (%)	
Improved/occluded	17 (63.0)
Stable	7 (26.0)
Worsened	3 (11.1)
Average days from FD placement to short-term angiographic results	3.93
Last follow-up angiographic results, no. (%)	
Occluded	11 (84.6)
Improved	1 (7.7)
Unchanged	1 (7.7)
Average months from FD placement to the last follow-up angiographic results	10.5

### Aneurysm re-rupture

**Figure 2.**
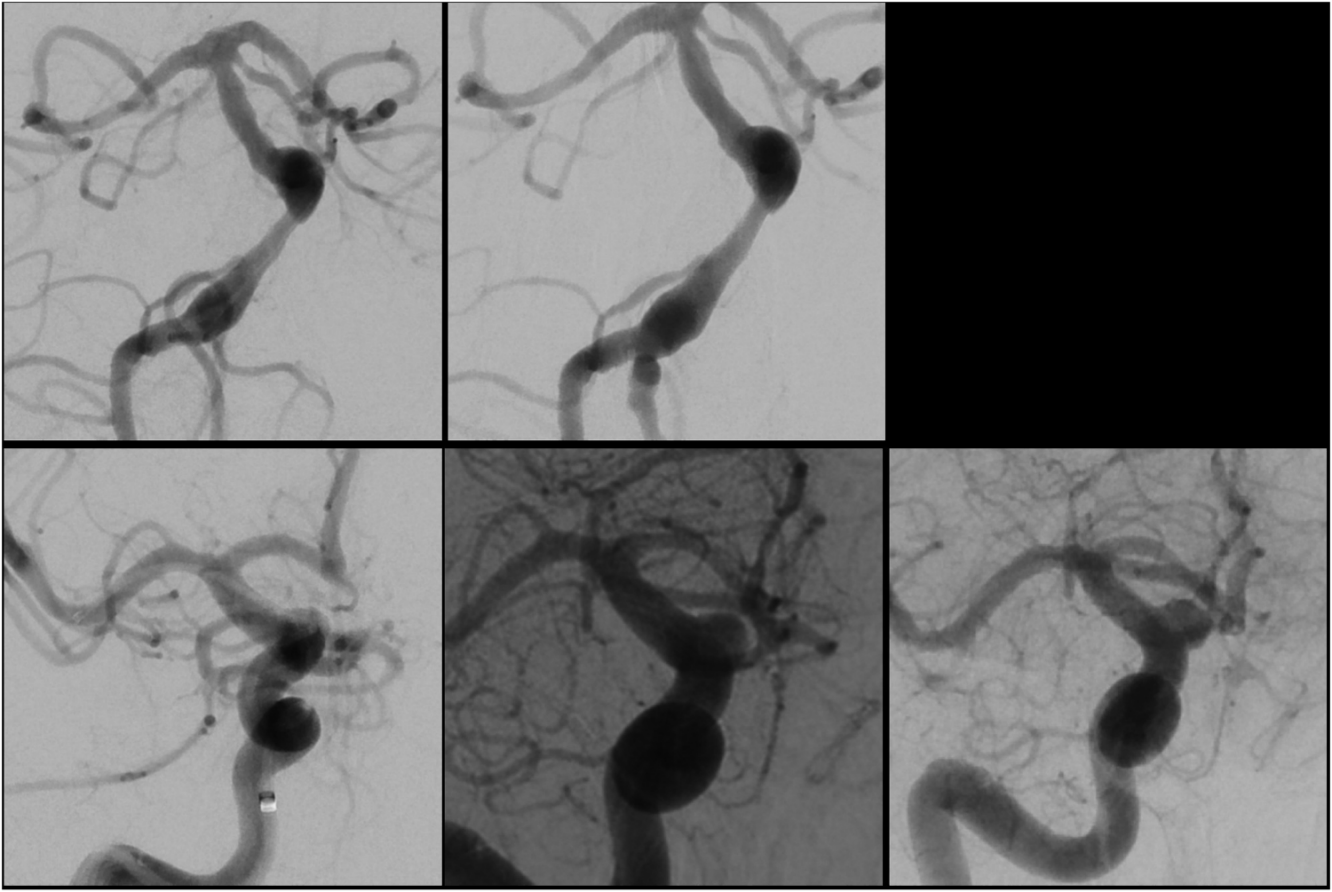
DSAs for re-ruptured aneurysm. (Top row left): Pre-treatment DSA of dissecting basilar aneurysm. (Top row right) Immediately post-FD treatment. (Bottom row left) Pre-treatment DSA of the right ICA ophthalmic segment dissecting aneurysm. (Bottom row middle) DSA 6 days post-FD placement showing slight enlargement of aneurysm. (Bottom row right) DSA 8 days post-FD placement showing clear enlargement of the aneurysm.

DSA: diagnostic subtraction angiography; FD: flow diverter; ICA: ICA: internal carotid artery.

Two patients (7.1%) suffered from re-rupture of their treated aneurysm. The first patient had a history of clival chondrosarcoma who underwent transsphenoidal resection followed by proton beam therapy 11 years prior to presentation. The patient presented as a Hunt and Hess (HH) grade II, modified Fisher 1 SAH. DSA showed a large dissecting aneurysm of the mid basilar artery with multiple blebs and irregularities (Figure 2). The patient was loaded with dual antiplatelet therapy (DAPT) and underwent placement of an FD with no immediate change in aneurysm filling. Nine hours after the procedure, they developed an acute headache, worsening mental status, and a new computed tomography scan showed diffuse SAH. An external ventricular drain (EVD) was placed, and an emergent DSA was done with placement of a second FD. No additional DSA was performed, and the patient was discharged to rehab on PBD26.

The second aneurysm re-rupture occurred in a patient who presented initially as a HH 4, modified Fisher 3 SAH from a dorsal ophthalmic segment dissecting aneurysm (Figure 2) that was treated with FD on PBD 0. The patient underwent two subsequent DSAs on PBD 5 and 7 due to vasospasm. In retrospect, there was progressive enlargement of her aneurysm that was not re-treated. It ultimately re-ruptured on PBD 10, and the patient was thereafter in a persistent vegetative state until passing away. Clinical outcomes for all 28 patients are summarized in Table 3.

**Table 3.** Clinical outcomes.

Aneurysm re-ruptures	2
EVD-related hemorrhages	5
Patients with EVDs	24
EVD management	
Removed bedside	17
Converted to VPS	4
Died prior to EVD removal	3
VPS-related hemorrhages	0
Other hemorrhagic complications	2
FD occlusion/stenosis	3
FD-related perforator strokes	2
Discharge disposition	
Home	12
Rehab	8
LTACH	2
SNF	3
In patient mortality	3
mRS at discharge, median	3

EVD: external ventricular drain; VPS: ventriculoperitoneal shunts; FD: flow diverter; LTACH: Long-Term Acute Care Hospital; SNF: Skilled Nursing Facility; mRS: modified Rankin Scale.

### Flow diversion treatment failure without re-rupture

**Figure 3.**
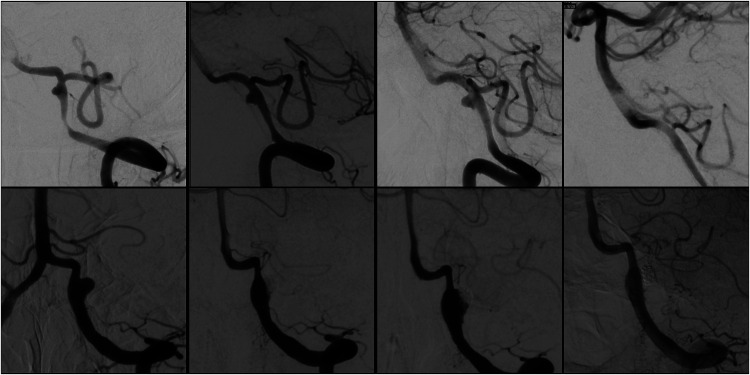
DSAs for treatment failure patients without re-rupture. (Top row left) Pre-treatment DSA of dissecting left vertebral artery aneurysm. (Top row middle left) 4 days after initial FD treatment. (Top row middle right) 12 days after initial FD treatment, the second FD was placed. (Top row right) 59 days after initial FD treatment. (Bottom row left) Pre-treatment DSA of dissecting left vertebral artery aneurysm. (Bottom row middle left) 4 days after initial treatment with coiling and FD placement. (Bottom row, middle right) 10 days after initial treatment, the second FD was placed. (Bottom row right) 14 days after initial treatment.

DSA: diagnostic subtraction angiography; FD: flow diverter.

Initial FD treatment failure without re-rupture was found in two patients (7.1%) on subsequent in-hospital DSA. The first patient presented as a HH 4 modified Fisher 3 SAH from a left V4 segment vertebral artery dissecting aneurysm treated with a single FD device on PBD 2. Follow-up DSA on PBD 5 showed mildly increased aneurysm size, but no re-treatment was done. On PBD 12, an additional DSA was done to evaluate vasospasm, which showed clear enlargement of the aneurysm, and a second FD was placed without complication and showed a reduction in aneurysm opacification. Follow-up outpatient DSA done, PBD 59 showed complete occlusion of the aneurysm (Figure 3).

The second was a patient who presented with a HH 2 modified Fisher 3 SAH from a dissecting V4 segment vertebral artery aneurysm initially treated with FD and coiling on PBD 0. On PBD 4, a routine follow-up DSA was done that showed new contrast filling around the FD device compared to the initial post-treatment runs. No treatment was done at that time. Another DSA on PBD 10 showed worsening dissection, and a second FD was placed with a subsequent reduction in aneurysm opacification. DSA done, PBD 14 showed persistent filling of the aneurysm but with improved occlusion and stagnation. No further DSA has been obtained for financial reasons, but at the 18-month follow up the patient had recovered well.

Two other patients had additional FDs placed on subsequent DSA. In both these cases, the aneurysms were unchanged on follow-up DSA, and the on-call neuro-interventionalist felt that the improved chance of obliteration was worth the risk of additional FD placement. One of these two patients suffered a basal ganglia stroke after the second FD treatment.

### Antiplatelet management

All patients were treated with DAPT after FD placement. Aspirin was included in the regimen for all 28 patients. As their second agent, 20 had clopidogrel, six had ticagrelor, and two had prasugrel. Sixteen patients were started on DAPT at least one day prior to FD, five were started on the morning of FD placement, and seven were started on DAPT intra-procedurally. Effect levels were checked within 24 to 48 h after FD, and the dosages or agents were adjusted accordingly. DAPT was continued for 6 months after FD placement, after which a single-agent antiplatelet agent was continued. DAPT was adjusted for procedures or complications based on the treating neurosurgeon's judgment.

Of the 24 EVDs placed, 12 were placed at least 24 h prior to the initiation of DAPT, and 10 were placed on the same day but prior to DAPT initiation. Two were placed after the initiation of DAPT. One patient had a lumbar drain placed the day before DAPT initiation.

Five patients required ventriculoperitoneal shunts (VPS) during their hospitalization. Operative technique and management of DAPT were not protocolized and depended on attending preference and clinical scenario. Aspirin was held for at most 2 days prior while clopidogrel/ticagrelor were held up to 4 days prior to the procedure, and both were restarted the following day. There were no VPS-related hemorrhagic complications.

### Hemorrhagic and ischemic complications

There were no significant intra-procedural complications; however, six patients (21.4%) had seven non-aneurysmal-related hemorrhagic complications after the initiation of DAPT. Out of 24 patients who had EVDs placed, five patients had EVD-related hemorrhages that were not present prior to FD treatment. Three were EVD tract hemorrhages that appeared after the initiation of DAPT, and 1 was a tract hemorrhage from an EVD placed after the initiation of DAPT. None of these required any intervention or cessation of DAPT. One patient had a small amount of intraventricular hemorrhage after multiple injections of intrathecal medications.

One patient developed a femoral artery pseudoaneurysm and associated hematoma from a DSA-related groin puncture that necessitated stenting and hematoma evacuation. Another patient underwent emergent hemicraniectomy on PBD0 from a ruptured dissecting ophthalmic artery aneurysm, followed by FD on PBD1 with initiation of DAPT. The following day, the patient declined due to the expansion of a previous intracerebral hemorrhage as well as a large epidural hematoma. The patient died on post-op day 13. Hemorrhagic complications are summarized in Table 3.

Five patients suffered ischemic complications after the placement of FD. Two patients had ischemic strokes of perforating branches covered by the FD. Three patients had in-stent stenosis or occlusion of their FD. The first had thrombosis of their FD 6 days after placement, causing aphasia, and underwent successful thrombectomy for re-canalization without any permanent neurological sequela. Brillinta, but not aspirin, had been held for 2 days at that time in anticipation of VPS placement. The second patient presented with symptomatic occlusion of their FD 1 month after stopping DAPT. She was taken for emergent thrombectomy with recanalization, but suffered several small scattered strokes and was discharged with persistent but improved dysarthria and weakness. The third patient had a small basal ganglia ischemic stroke the day after a second FD device was placed over a dissecting ophthalmic segment aneurysm. Emergent DSA was performed, showing in-stent stenosis that was angioplastied. Ischemic complications are summarized in Table 3.

### Long-term angiographic results

Thirteen patients had follow-up DSA >4 months after rupture. At the last follow-up, an average of 10.2 months, 11 patients (84.6%) showed complete obliteration, and one patient (7.7%) showed improved but persistent filling. One patient (7.7%) showed no change in aneurysm filling since initial FD placement. No additional aneurysm treatment was done for this patient at that time, as the repeat DSA was done for an acute basilar occlusion necessitating thrombectomy, and has not had any subsequent follow-up. Long-term angiographic results are shown in Table 2.

### Blood blister-like aneurysms

Four aneurysms met the strict criteria of dissecting dorsal wall aneurysms in the ophthalmic segment of the ICA, or blood blister-like aneurysms (Figure 1). One patient with a blood blister-like aneurysm died after aneurysm re-rupture on PBD10, as discussed in the “Aneurysm re-rupture” section of the results. A second patient died on PBD7 from the consequences of initial brain injury combined with a large post-op hematoma while on DAPT, as discussed in the “Hemorrhagic and ischemic complications.” The two other patients had their aneurysms successfully occluded by PBD1 and 4 months, respectively, as confirmed by DSA.

## Discussion

Dissecting aneurysms carry a significant risk requiring urgent treatment. Mizutani et al. studied 42 patients with ruptured vertebrobasilar dissecting aneurysm and found that 29 patients (69%) suffered re-rupture prior to treatment. Seventeen patients (56.7%) of these re-ruptures occurred within 24 h of initial rupture, and 24 patients (80%) occurred within 1 week.^15^ The treatment, however, remains controversial as clip reconstruction is not possible due to the friable wall, and primary coiling is limited due to wide necks and unstable walls. This leaves a gap in treatment options that could potentially be filled by FD.

A meta-analysis in 2018 by Cagnazzo et al. analyzed patients from 20 different studies and included 223 ruptured aneurysms of varying subtypes. They reported a combined angiographic occlusion rate of 32% immediately after FD deployment, and 89% at 6 to 12 months follow-up, with a total re-rupture rate of 4%.^16^ Cohen et al. have published the largest single-center review with 76 ruptured aneurysms and reported no target aneurysm re-ruptures. They report an immediate total/near-total occlusion rate of 14.5% after FD deployment and a 100% rate at 12-month follow-up.^17^

The literature pertaining to only ruptured dissecting or blister aneurysms is even more sparse and is composed exclusively of small case series. The largest of these was published by Bounajem et al. and includes 33 patients with intracranial ICA dissecting aneurysms. They report one intra-procedure rupture with no re-ruptures after FD placement. Of the 23 patients with follow-up imaging, 82.6% had complete occlusion at a mean follow-up of 3.49 months.^18^ A pooled analysis of 226 patients with dissecting aneurysms treated with FD showed a re-rupture rate of 2%. Immediate angiographic occlusion was roughly 10% in those treated with FD alone, and 70% in those treated with FD-assisted coiling. Occlusion at last follow-up was 87% and 98% in the FD and FD-assisted coiling groups, respectively, which are in keeping with the results found in our study.^12^

**Figure 4.**
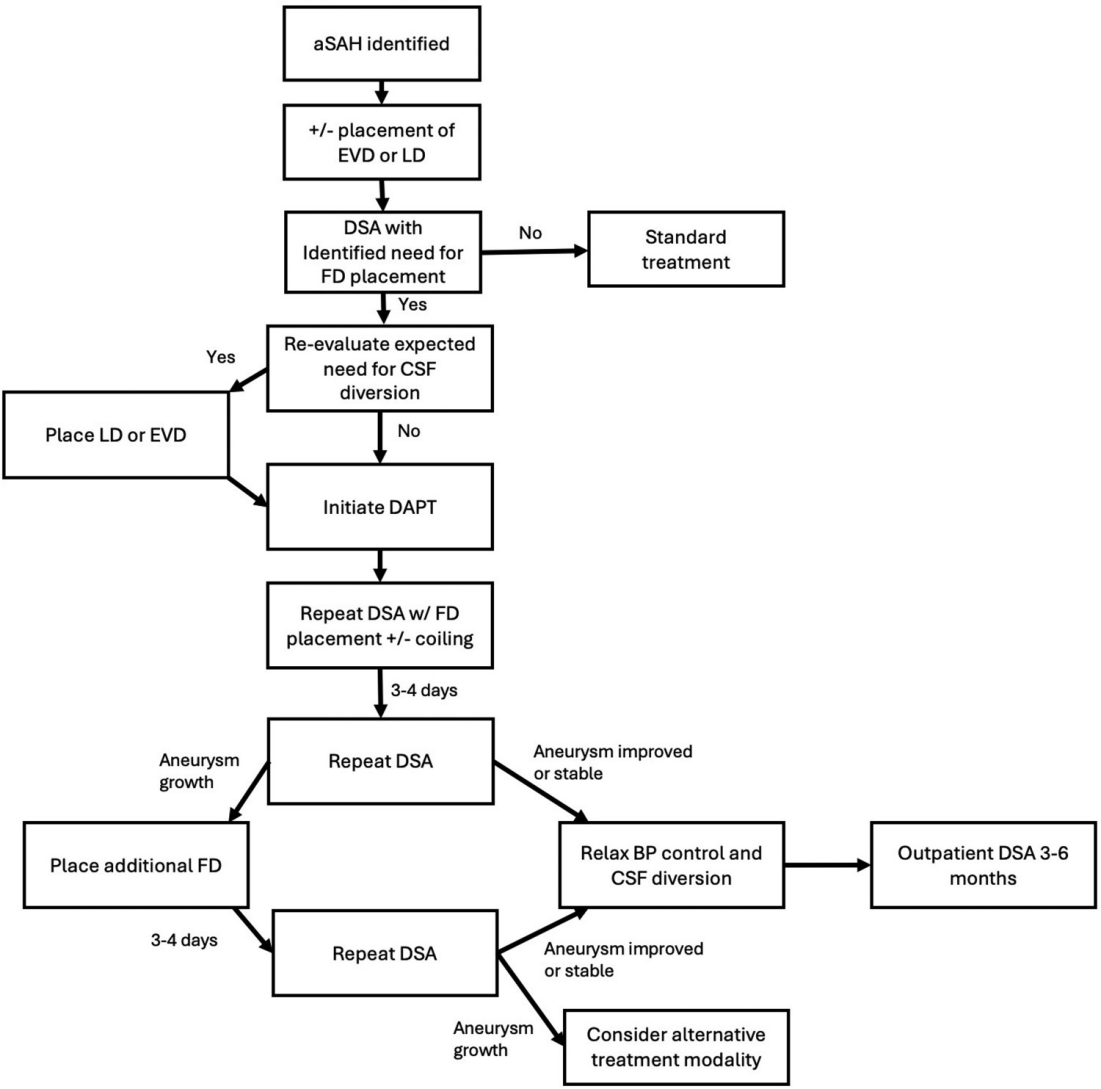
Flow diagram for proposed management algorithm.

Due to the inherently uncertain evolution of these aneurysms, combined with the fact that FD does not provide immediate and complete protection, we routinely use short-term DSA follow-up after FD (mean time of 3.9 days in this series). We found this strategy to be determinative for additional FD placement. In our series, four patients showed early treatment failure. One patient re-ruptured prior to the initial follow-up DSA (without opportunity for early DSA benefit), while the other three patients (one re-rupture, two aneurysm growth) showed evidence of aneurysmal enlargement on the initial follow-up DSA. Early DSA in these three cases was considered helpful to identify a high-risk subgroup of treated aneurysms that were continuing to grow with a single FD device. Based on this finding, we recommend early post-treatment DSA within three to four days to reassess the ruptured dissecting aneurysm and confirm that there is no new enlargement. Figure 4 shows a flowchart with our proposed algorithm and sequence for EVD or lumbar drain placement, initiation of DAPT, and DSA.

None of the 24 (89%) patients who had stable or improved DSA findings went on to re-rupture. Thus, an early angiogram confirming no growth may have some predictive value for “protection.” It is now our typical protocol to treat the aneurysm as secured if, after the initial follow-up DSA, the dissection is stable or improved. Correspondingly, if an aneurysm grows on the early angiogram, we believe these to be high risk and recommend adding an additional FD to prevent re-hemorrhage. As noted, all but three of the patients in our group were initially managed with a single FD device. It may be that certain dissecting aneurysms may benefit from two FD devices at the time of initial treatment, but this cannot be evaluated in our study, as we have an insufficient number of patients treated in this manner to try to draw any conclusions. Additionally, the decision-making behind the placement of multiple devices upfront was not protocolized.

Although useful for these difficult-to-manage aneurysms, FD in the acute SAH setting has moderate risks and should be weighed against other options such as vessel sacrifice, bypass, or clip wrapping. Our reported hemorrhagic and ischemic complications are in line with those of prior published series.^16^ There were seven hemorrhagic complications amongst the 28 patients, five of which were EVD related, and one had a significant clinical impact.

## Conclusions

The use of FD for ruptured dissecting aneurysms with or without coiling is a reasonable option for these technically challenging lesions. However, the re-rupture rate and associated DAPT complications are significant. To mitigate the risk of re-rupture, we recommend repeat routine angiography three to four days after FD to confirm aneurysm stability. Those with aneurysmal morphology changes should be considered for an additional FD.
